# Blade Plate With Autogenous Bone Grafting to Salvage Peri Ankle Nonunions

**DOI:** 10.1177/10711007231165303

**Published:** 2023-04-28

**Authors:** Mees Paulus Emmelot, Robert Kaspar Wagner, Stein Jasper Janssen, Peter Kloen

**Affiliations:** 1 Department of Orthopedic Surgery and Sports Medicine, Amsterdam UMC location Meibergdreef, Amsterdam, the Netherlands; 2 Amsterdam Movement Sciences, Musculoskeletal Health, Amsterdam, the Netherlands

**Keywords:** blade plate, nonunion, ankle, salvage, pseudoarthrosis, graft

## Abstract

**Background:**

Salvage surgery for a nonunion around the ankle is challenging. Poor bone stock, stiffness, scarring, previous (or persistent) infection, and a compromised soft tissue envelope are common in these patients. We describe 15 cases that underwent blade plate fixation as salvage for a nonunion around the ankle, including patient/nonunion characteristics, Nonunion Scoring System (NUSS), surgical technique, healing rate, complications, and long-term follow-up with 2 patient-reported outcome measures.

**Methods:**

This is a retrospective case series from a level 1 trauma referral center. We included all patients that underwent blade plate fixation for a long-standing nonunion of the distal tibia, talus, or failed subtalar fusion. All patients had autogenous bone grafting, including 14 with posterior iliac crest grafts and 2 with femoral reamer irrigator aspirator grafting. Median follow-up was 24.4 months (interquartile range [IQR], 7.7-40). Main outcome measures were (time to) union, and functional outcomes using the 36-item Short Form Health Survey (SF-36) physical component summary (PCS) and mental component summary (MCS), and the Foot and Ankle Outcome Score (FAOS).

**Results:**

We included 15 adults with a median age of 58 years (IQR, 54-62). The median NUSS score at the time of index surgery was 46 (IQR, 34-54). Union was achieved after the index procedure in 11 of 15 patients. Additional surgery was performed in 4 of 15 patients. Union was achieved in all patients at a median of 4.2 months (IQR, 2.9-11). The median score for the PCS was 38 (IQR, 34-48, range 17-58, *P* = .009), for the MCS 52 (IQR, 45-60, range 33-62, *P* = .701), and for the FAOS 73 (IQR, 48-83).

**Conclusion:**

In this series, our use of blade plate fixation with autogenous grafting was an effective method for managing a nonunion around the ankle allowing for alignment correction, stable compression and fixation, union, and fair patient-reported outcome scores.

**Level of Evidence:**

Level IV, therapeutic.

## Introduction

A nonunion around the ankle is poorly tolerated and has a substantial impact on health-related quality of life.^5,17,26^ Symptoms include limited weightbearing ability, pain, and (progressive) deformity. The physician is often searching for the “ideal” salvage. In some cases, amputation may seem the only reasonable option.^2^ Fortunately, for many patients, reproducible options for customized salvage exist. Surgical techniques used for nonunion in this region include plating, external fixation, bone transport, (vascularized) bone grafting, and arthroscopically assisted fixation both at the distal tibia and/or at the subtalar level.^2,15,25,28^

We consider the 95-degree condylar blade plate (“blade plate”) a reliable technique with few complications for nonunion treatment of the proximal humerus, proximal/distal femur, and the ankle. With introduction of (anatomic) locking plates and more advanced intramedullary nails, the blade plate is becoming obsolete for younger surgeons. We believe the blade plate still has unique properties at very low cost and should remain in the orthopaedic trauma surgeon’s armamentarium. Therefore, our aim is to present our technique and experience with using a blade plate as a salvage option for a nonunion around the ankle. We describe patient and nonunion characteristics, previous surgeries, Nonunion Scoring System score, surgical technique, complications, and long-term follow-up with 2 validated patient-reported outcome scores.^6^

## Material and Methods

All patients that underwent a salvage procedure for a nonunion around the ankle using a blade plate in our level 1 tertiary care trauma center were identified in the senior author’s prospectively kept database. All surgeries were performed by a fellowship-trained orthopaedic trauma surgeon with experience in nonunion surgery. We identified 15 patients operated between 2004 and 2021 that met the following inclusion criteria: (1) nonunion of the distal tibia, talus, or failed subtalar fusion after previous surgical treatment, (2) surgical treatment with blade plate, and (3) aged 18 years or older.

Ethical approval was waived by the local Medical Ethics Review Committee (reference W21_138 # 21.153). Baseline, surgical, and clinical outcome variables were collected from the electronic medical records.

No patient was lost to follow-up before healing. Patient-reported outcome data were available for 13 of 15 patients at a median of 7.1 years (IQR, 2.6-8.0) after surgery. One patient did not participate, and 1 had passed away.

### Surgical Technique and Indications

The blade plate was indicated for a nonunion of the distal tibia, talus, or failed subtalar fusion with poor bone stock. All patients received a preoperative CT scan. No patient had elevated infection parameters (erythrocyte sedimentation rate, C-reactive protein, or white blood cell count; assessed in 13 of 15 patients), signs of infection (redness, draining sinus, or wound healing defect), or received antibiotics in the 14 days prior to surgery. Prior to surgery, we customized 1 or 2 blade plates by cutting the blade length based on preoperative plans.

For iliac crest bone grafting (14 of 15), patients were positioned prone. Femoral bone graft (2 of 15) was harvested retrograde using the reamer irrigator aspirator in a lateral decubitus position.

The surgical approach depended on previous treatment(s), soft tissue conditions, and nonunion location. In case of a soft tissue defect or previous soft tissue reconstruction, a plastic surgeon with expertise in microsurgery was consulted preoperatively.

A thigh tourniquet was used. The approach was a single incision: midline posterior in 7 of 15, anterolateral in 4 of 15, and lateral in 4 of 15 patients.

**Figure 1.**
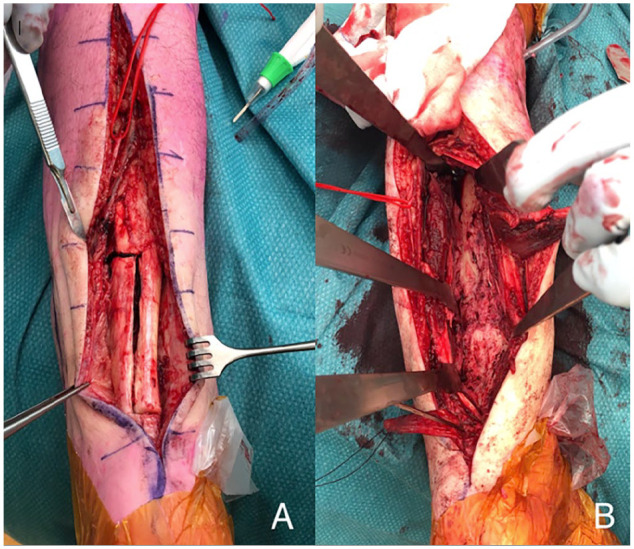
Case 15 in Table 1. Z-shaped tenotomy of the Achilles tendon for exposure of the distal tibia using a posterior approach (A, B).

For the posterior approach (7 of 15), the patient was positioned prone and a longitudinal midline incision was made over the Achilles tendon. The neurovascular bundle and flexor hallucis longus were identified and retracted medially. A Z-shaped tenotomy of the Achilles tendon was performed (Figure 1A). For a stiff nonunion, the AO-femoral distractor can help distract and align the nonunion, tibiotalar, or tibiotalocalcaneal joint.

For a lateral or anterolateral approach (8 of 15), the patient was positioned supine. For the anterolateral approach the incision was centered between the tibia and fibula, and for the lateral approach it was centered over the fibula. Incision was usually 15 cm.

Following the approach, all hardware was removed. Five deep tissue samples and 1 screw were sent for culture. Subsequently intravenous cefazoline was given. The nonunion site was thoroughly debrided until viable bleeding nonunion ends and bleeding cortical bone was seen (Figure 1B).

**Figure 2.**
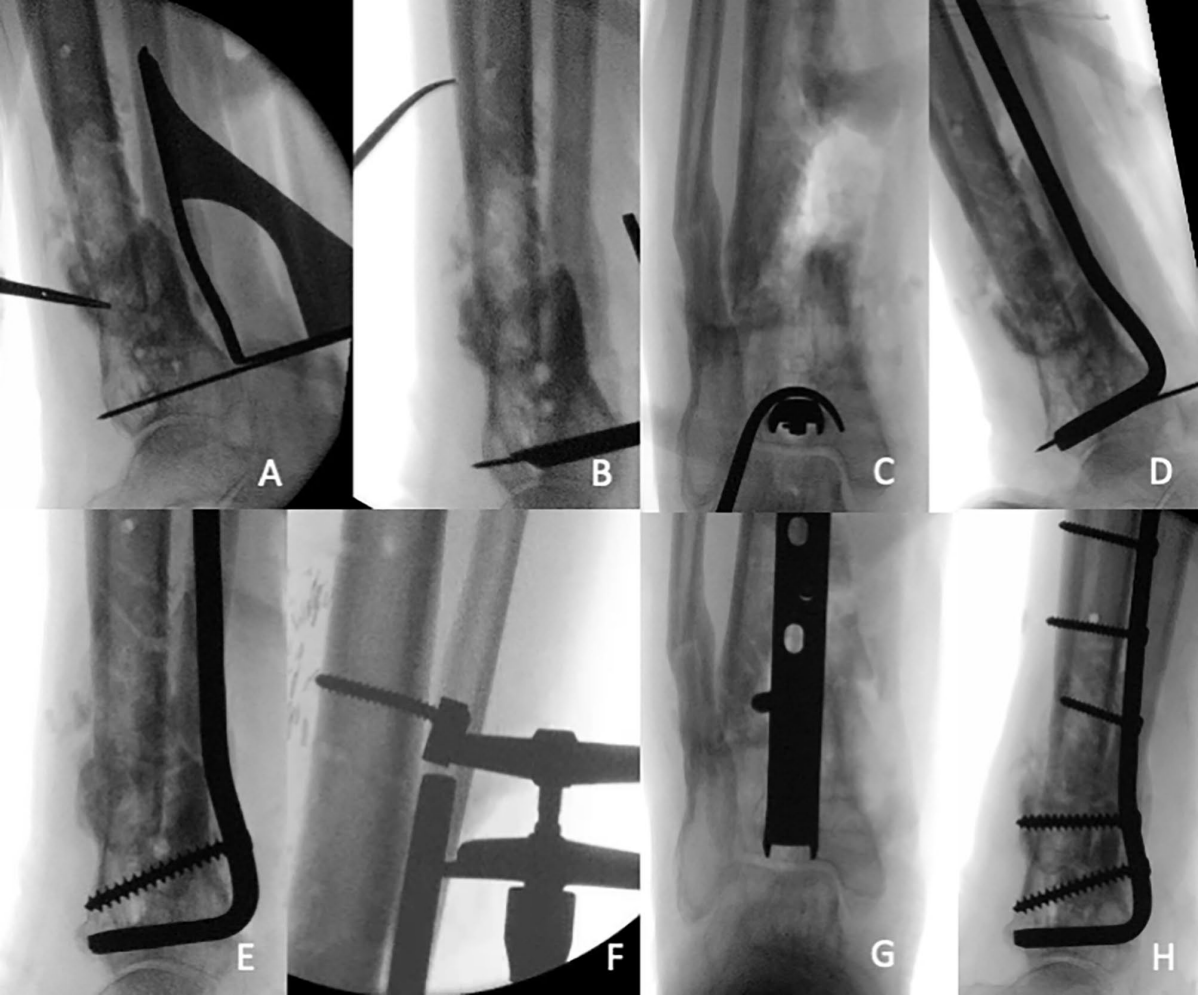
Case 15 in Table 1: Intraoperative fluoroscopy imaging of blade plate placement in the left ankle. (A) The 95-degree condylar plate guide is placed along the cortex of the distal tibia and a K-wire is inserted. (B) The chisel guide is placed against the K-wire. Using the guide, the chisel is hammered until desired depth. (C) The chisel position is checked radiologically (a bone hook around the chisel is used to get a bull’s eye anteroposterior view). (D) The chisel is then removed and exchanged for the blade plate. (E) The blade plate is fixated distal of the nonunion with one or two screws. (F) The AO-tensioner device is fixated proximally for compression (notice the deformation of the screw). Final alignment in (G) anteroposterior and (H) lateral view.

**Figure 3.**
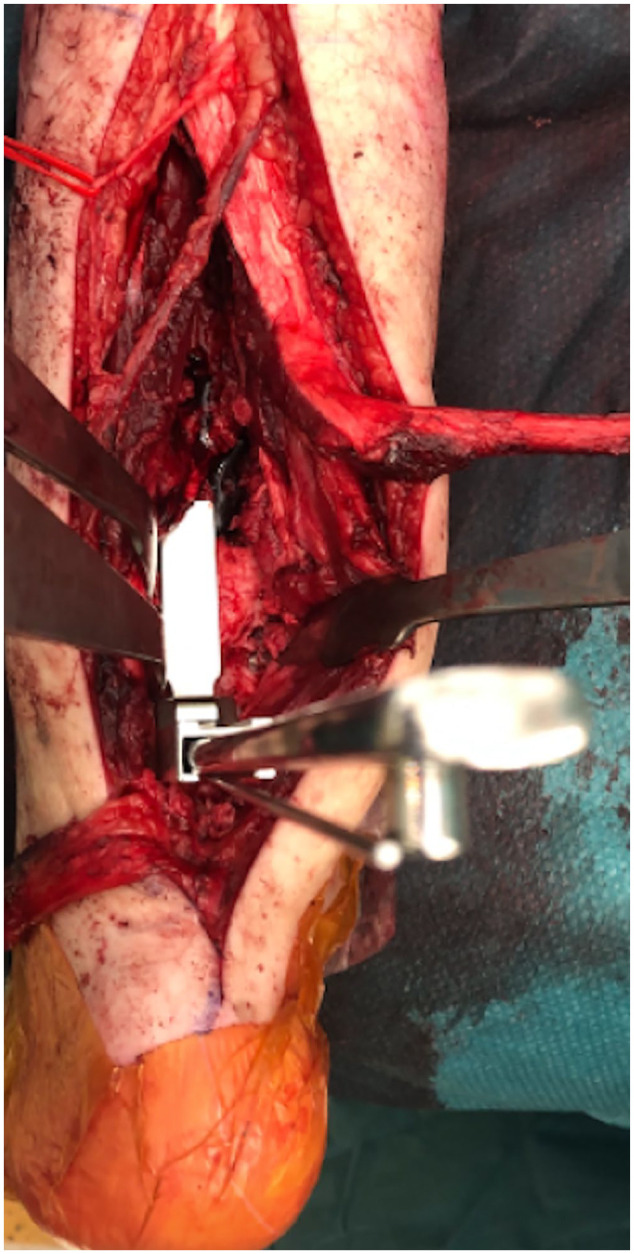
Case 15 in Table 1: Intraoperative view of the blade plate seating chisel creating a path for the blade plate in the distal tibia.

**Figure 4.**
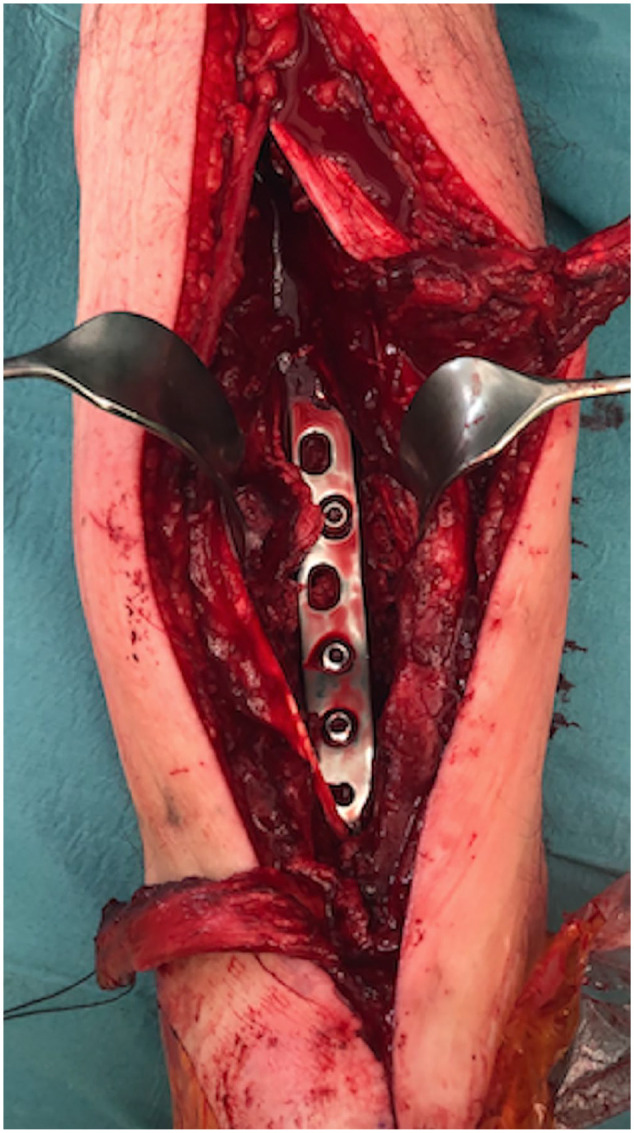
Case 15 in Table 1: Intraoperative view after placement of the 95-degree condylar blade plate in the distal tibia.

Depending on the indication, the blade plate position was in the talus, calcaneus, or distal tibia. First, the condylar blade plate guide was placed along the bone at the level of the planned entry. A K-wire is placed under fluoroscopic guidance (Figure 2A). For the distal tibia, we went as distal as possible because the bone quality was the least compromised there. The ideal position is parallel to the joint in the lateral view and midline in the anteroposterior view. The ideal fluoroscopic view may need adjustment of exo- or endorotation of the leg. Once the guiding K-wire was in perfect position, the pathway for the blade was prepared using the chisel and chisel guide as directed by the K-wire. The chisel was gently hammered into the bone under fluoroscopic guidance (Figure 2B and 3). Once the chisel was in appropriate position (Figure 2C), and if the goal was an arthrodesis, the tibiotalar and/or the talocalcaneal joint were opened by gently pulling on the chisel (or distracting the large AO-femoral distractor if placed) and residual articular cartilage was removed with a curette until bleeding subchondral bone was seen. Some bone graft and/or DBM was then placed between the denuded joint surfaces. The chisel was exchanged for the blade plate (Figure 2D). There always was a (near) perfect fit of the blade without having to re-contour the plate. The blade plate was then fixated distal of the nonunion/arthrodesis site using 1 or 2 screws (preferably cortical 4.5-mm, but sometimes a fully threaded cancellous large-fragment screw) aiming toward the tip of the blade (Figure 2E). Depending on the deformity, the proximal part of the plate (with the holes) may not align yet with the tibia. This was easily corrected to (near) perfect by manually aligning the ankle to a neutral position. The AO-articulated tensioner device was used to fine-tune the reduction while providing compression with care to prevent cutout of the blade (Figure 2F). The blade plate was then fixated proximal of the nonunion/arthrodesis site (Figure 2G and 2H, and Figure 4). Bone graft was added. In 1 patient, fibular autograft was combined with iliac crest bone grafting and reamer irrigator aspirator. In 7 of 15 patients, autologous bone graft was combined with demineralized bone matrix putty (DBX; DePuy Synthes, Amersfoort, the Netherlands). The Achilles tenotomy was closed anatomically and the wound was closed in layers.

Final reconstructions were a tibiotalar arthrodesis in 6 of 15, tibiotalocalcaneal arthrodesis in 3 of 15, and distal tibial fixation in 6 of 15. Blade plates used were as follows: 95-degree condylar in 8 of 15, adolescent 95-degree condylar in 6 of 15 patients, and a cannulated blade plate in 1 patient (all from DePuy Synthes, Amersfoort, the Netherlands). The customized blade had a median length of 40 mm (IQR, 35-40) and a median number of 9 shaft holes (IQR, 8-12).

Postoperatively, all patients received a splint for 1-2 days, after which a nonweightbearing short-leg cast was applied for 6 weeks. Antibiotics were given until culture results at 14 days were negative.

### Outcome and Explanatory Variables

Baseline variables collected included age and sex, mechanism of injury, date of injury, fracture configuration using OA/OTA classification,^21^ and Gustilo-Anderson Classification system,^18^ requirement of ambulatory devices, and previous interventions. The nonunion was described using the Weber and Çech classification^30^ and the Nonunion Scoring System.^6^ The diagnosis of infection was based on positive intraoperative cultures, purulent drainage, and clinical inspection.^22^

The index surgery was defined as the first nonunion surgery with a blade plate at our institution. Surgical variables collected included approach (posterior, lateral, or anterolateral), procedure type, use of bone graft, blade plate characteristics (length, volume, holes), screw (type, number, length, volume), and screw-hole ratio (number of screws divided by the number of plate holes). Our medical technical department calculated the volume of blades and screws inserted, using computer modeling.

### Clinical Outcome Variables

Clinical outcome variables included time to union defined as the time from the index surgery until bony bridging of at least 3 of 4 cortices, union rate, additional treatments (antibiotic treatment or removal of hardware) or surgeries after our index surgery to procure healing, and complications. Clinical and radiographic follow-up was obtained at 2 and 6 weeks, 3 and 6 months, and 1 year after index surgery or until union. Treatment was considered as failed in case of persistence of infection, nonunion, absence of soft tissue healing, or need for amputation.

### Patient-Reported Outcome Measures

Patient-reported outcomes were measured using the 36-Item Short Form Health Survey (SF-36) and the Foot and Ankle Outcome Score (FAOS).^4,19,20^ The SF-36 measures the physical component summary (PCS) and a mental component summary (MCS). Higher SF-36 scores represent better physical or mental health (range 0-100), and higher FAOS scores better functionality and quality of life (range 0-100).

### Statistical Analysis

Numerical variables are described as median with interquartile range (IQR). Categorical variables are presented as frequencies with percentages. Cases with missing values are removed (complete case analysis). The PCS and MCS scores of the SF-36 are tested against the normative Amsterdam population average (PCS 50, MCS 49) using the nonparametric signed-rank test.^1^ Statistical analyses were performed using IBM SPSS Statistics, version 28.0 (IBM, Armonk, NY, USA).

## Results

### Baseline and Surgical Variables

There were 12 males and 3 females with a median age of 58 years (IQR, 54-62). Eleven patients had suffered a high-energy trauma: motor vehicle accident (n = 7), crush injury (n = 1), or fall from height (n = 3) (Table 1). Two patients suffered a low-energy trauma (fall from standing height). One patient had a failed tibiotalar arthrodesis and 1 patient a failed supramalleolar closing-wedge osteotomy. Median duration from initial injury until index surgery was 16 months (IQR, 11-22). Fractures were classified as open in 4 of 15 patients (Gustilo I [n = 1], II [n = 2], IIIA [n = 1]). Before index surgery, all patients required assistive devices such as crutches (n = 13) or wheelchair (n = 2) for ambulation.

**Table 1.** Individual Patient Data Blade Plate Cases.

Case	Age at Injury (y)	Sex	Side	Gustilo	AO/OTA	W&Ç	NUSS	Pre-OP Malalignment	Previous Infection	No. of Prior Surgeries	Previous Procedures	Duration of Nonunion (mo)	Approach	Procedure	Plate Type (Blade Length)	Screw/ Hole Ratio	No. of Screws	Additional Bone Graft	Bacterial Species Cultured	Index Surgery Until Union (mo)	Follow-up (mo)	Complications	Additional Surgeries
1	55	Male	Left	Closed	812A	OT	34	5 degrees varus	No	3	(1) TTA with screws (2) TTCA with plate (3) Screw removal	17.1	Lateral	TTCA	95-degree adolescent condylar blade plate (40mm), 7 holes	0.6	4	ICBG	None	2.8	2.8	None	None
2	68	Male	Left	Closed	43A1	AT	54	3 degrees varus	Yes	8	(1) DT plate fixation (2) Hardware removal (3) External fixation (4) Plate fixation (5) ROH + external fixation + gentamycin beads (6) Debridement + adjustment external fixation + new gentamycin beads (7) Gracilis free flap (8) Plate fixation + fibula allograft + bone graft	52.8	Posterior	TTCA	95-degree condylar blade plate (60mm), 12 holes	0.5	6	ICBG + 5 mL DBM	None	35.2	70.1	None	Blade plate exchange + ICBG
3	54	Male	Right	Closed	43A1	HT	34	7 degrees valgus, 35 degrees antecurvatum	Yes	1	(1) Plate and screw fixation	29.6	Lateral	DT fixation	90-degree cannulated blade plate (40mm), 6 holes	0.8	5	ICBG	None	13.1	70.2	None	(1) ICBG + 3.5 LCP (2) ROH
4	58	Male	Left	Closed	43C1	OT	46	5 degrees varus, 5 degrees exorotation	Yes	4	(1) DT screw fixation (2) ROH + external fixation (3) Addition external fixation + gentamycin beads (4) Split skin graft	15.9	Posterior	TTA	95-degree adolescent condylar blade plate (50mm), 7 holes	0.6	4	ICBG + 5 mL DBM	None	11.1	17.8	None	ICBG + 3.5 LCP
5	44	Male	Left	Grade I	43C2	OT	54	10 degrees varus	Yes	3	(1) External fixation (2) TTA with plate + bone graft (3) ROH + debridement + external fixation	45.4	Posterior	TTA	95-degree condylar blade plate (50mm), 9 holes	0.6	5	ICBG	None	38.2	38.2	None	Blade plate exchange + ICBG
6	71	Male	Right	Closed	43C1	OT	48	3 degrees valgus	Yes	2	(1) Intramedullary nailing (2) ROH + external fixation	10.5	Posterior	TTA	95-degree adolescent condylar blade plate (40mm), 7 holes	0.6	4	ICBG	None	3.1	42.0	None	None
7	54	Male	Right	Closed	–	HT	34	6 degrees valgus	Yes	3	(1) Ankle arthroscopy (2) TTA with screws (3) TTCA with screws	50.1	Lateral	TTCA	95-degree adolescent condylar blade plate (40mm), 7 holes	0.6	4	ICBG	None	2.8	5.8	None	None
8	61	Male	Right	Closed	43C3	OT	64	2 degrees varus, 14 degrees recurvatum	Yes	4	(1) DT plate fixation (2) ROH + external fixation (3) Gracilis free flap (4) Plate fixation + bone graft	17.7	Anterolateral	DT fixation	95-degree condylar blade plate (40mm), 9 holes	0.6	5	RIA femur	None	4.3	53.5	Screw breakage (3)	None
9	60	Female	Right	Closed	43C3	OT	54	4 degrees varus	Yes	1	(1) DT plate fixation	25.6	Posterior	TTA	95-degree condylar blade plate (40mm), 13 holes	0.3	4	ICBG	*Staphylococcus epidermidis*	2.6	26.2	None	None
10	47	Male	Left	Closed	–	OT	30	4 degrees varus	No	3	(1) Arthroscopically assisted TTA with screws (2) TTA with additional screws (3) Screw removal	10.8	Posterior	TTA	95-degree condylar blade plate (40mm), 9 holes	0.4	4	ICBG	None	4.2	29.1	None	ROH
11	58	Male	Left	Closed	43C2	OT	34	22 degrees valgus	Yes	3	(1) External fixation (2) DT plate fixation (3) Latissimus dorsi free flap	22.8	Anterolateral	DT fixation	95-degree adolescent condylar blade plate (30mm), 9 holes	0.6	5	ICBG + 10 mL DBM	None	6.9	24.4	Sensibility disorder	None
12	62	Male	Left	Grade II	42A1	HT	40	10 degrees varus, 10 degrees recurvatum	Yes	1	(1) Plate fixation	27.2	Lateral	DT fixation	95-degree condylar blade plate (30mm), 15 holes	0.6	9	ICBG + 5mL DBM	*Cutibacterium acnes*	1.9	6.9	None	None
13	48	Female	Left	Closed	42A1	OT	46	14 degrees valgus, 12 degrees recurvatum	No	2	(1) Intramedullary nailing (2) ROH + DT plate fixation + bone graft	18.7	Anterolateral	DT fixation	95-degree condylar blade plate (30mm), 14 holes	0.5	7	ICBG + 10 mL DBM	*Cutibacterium acnes*	4.9	12.3	None	None
14	70	Female	Right	Grade IIIA	43C3	OT	62	None	Yes	5	(1) External fixation (2) Revision external fixation (3) Osteomyelitis debridement + propellor flap + gentamycin beads + adjustment external fixation (4) Illizarov frame + Masquelet + bone graft (5) Debridement + adjustment frame	22.9	Anterolateral	TTA	95-degree adolescent condylar blade plate (30mm), 9 holes	0.7	6	ICBG + RIA femur + 10 mL DBM + fibula autograft	None	3.1	3.1	None	None
15	57	Male	Left	Grade II	43A3	OT	66	1 degrees varus, 4 degrees recurvatum	Yes	8	(1) External fixation (2) Intramedullary nailing + split skin graft (3) ROH + external fixation (4) Intramedullary nailing + bone graft (5) ROH + metaphyseal LCP as external fixation (6) Ilizarov frame + bone graft (7) Ilizarov frame adjustment (8) Additional debridement + bone graft + metaphyseal LCP	32.8	Posterior	DT fixation	95-degree condylar blade plate (40mm), 12 holes	0.6	7	ICBG + 5cc DBM	*Staphylococcus caprae*, *Staphylococcus capitis*	3.4	9.6	None	None

Abbreviations: AO, arbeitsgemeinschaft für osteosynthesefragen; AT, atrophic; DBM, demineralized bone matrix; DT, distal tibial; HT, hypertrophic; ICBG, iliac crest bone grafting; LCP, locking compression plate; NUSS, Nonunion Scoring System; OT, oligotrophic; OTA, Orthopaedic Trauma Association; RIA, reamer irrigator aspirator; ROH, removal of hardware; TTA, tibiotalar arthrodesis; TTCA, tibiotalocalcaneal arthrodesis; W&Ç, Weber&Çech.

**Figure 5.**
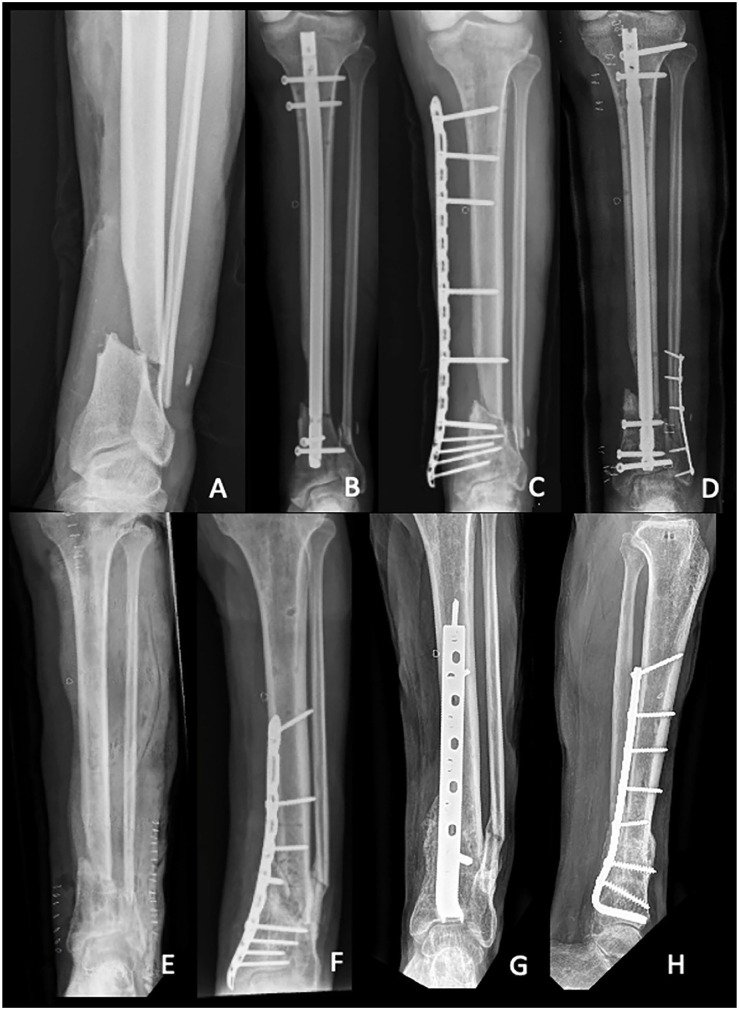
Case 15 in Table 1: (A) This patient had a high-energy forklift crush injury Gustilo grade II distal cruris fracture, OA grade 43A3. This patient had 8 surgeries previous to blade plate fixation (of which a selection is shown in the following images). (B) Primarily the patient received external fixation (not shown), followed by intramedullary nailing in suboptimal alignment. One month later, the patient developed a deep infection. (C) We removed the nail, took cultures, and applied a metaphyseal locking compressing plate as an external fixator. (D) After treating the infection, we removed the external fixator plate 4 months later and placed a new intramedullary nail. (E) Unfortunately, 6 months later, there were signs of deep infection, requiring hardware removal and debridement. The patient received a short-leg cast and the infection was treated with antibiotics. Two months later, an Ilizarov frame was placed with additional autologous bone grafting (not shown). Six months later, the frame was exchanged for a short-leg cast because it was intolerable (not shown). Three months later, fixation of the distal tibia was performed using a metaphyseal locking compression plate with iliac crest bone graft. (F) However, union was not achieved. Six months later (33 months after the initial injury), we performed revision fixation with a blade plate. (G, H) Union was achieved 3.4 months later.

**Figure 6.**
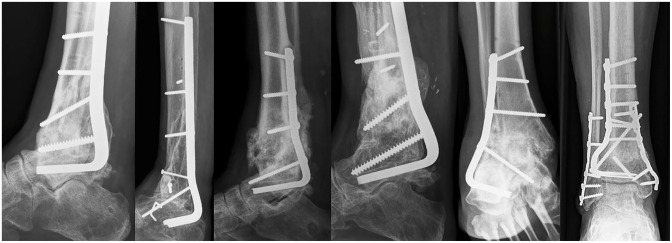
Final follow-up radiographs of 6 representative cases (4 posterior and 2 lateral approaches). These are cases 2, 9, 4, 5, 7, and 3, respectively, from Table 1. Only the view perpendicular to the blade is shown.

The median Nonunion Scoring System score at the time of index surgery was 46 (IQR, 34-54), classifying the cohort as “complex” requiring specialized care and treatment.^6^ In our series, 12 of 15 patients had a history of fracture-related infection and a median of 3 (IQR 2-4) previous surgeries. The patient (case 15) shown in Figures 1 to 4 had undergone a total of 8 previous surgeries before the blade plate salvage was done (Figure 5). There were 7 of 15 patients who had previous osteosynthesis with bone graft. Before index surgery, 6 of 15 patients had undergone soft tissue coverage, including a propellor flap (n = 1), vascularized free flap (m. gracilis [n = 2] or m. latissimus [n = 1]), or an autologous split skin graft (n = 2). Malalignment of the nonunion was present in 11 of 15 patients (Table 1). Figure 6 shows a representative sample of the patients.

Volume calculations demonstrated that a total of 2621 mm^3^ (IQR, 2277-3352) metal was inserted into the bone distal from the nonunion/arthrodesis site. Nine patients were previously operated with locking compression plate fixation, with a total volume of 2616 mm^3^ (IQR, 1711-3063) metal inserted distal from the nonunion/arthrodesis site (*P* = .340).

### Clinical Outcome Variables

Union was achieved in 11 patients after the index procedure. In 4 patients, additional surgery was necessary to obtain union. After revision surgery, union was achieved in all patients after a median of 4.2 months (IQR, 2.9-11). Median follow-up was 24.4 months (IQR, 7.7-40). In 9 of 15 patients, a CT was performed to confirm consolidation. Postoperative alignment was neutral in 14 of 15 patients. One patient had residual antecurvatum of 17 degrees (preoperative antecurvatum was 35 degrees). Five patients required customized footwear for a median postoperative leg-length discrepancy of 2.4 cm (IQR, 2.2-2.6). Despite complete consolidation, 1 patient still used crutches for long distances because of leg weakness, and 1 patient was wheelchair-bound at most recent follow-up. Because of the COVID-19 pandemic, he had not been able to participate in a formal physical therapy program. He has since started an intensive rehabilitation program and is improving rapidly.

### Culture Results From Index Surgery

Four patients had positive intraoperative cultures. Three of these had a history of fracture-related infection (Table 1). The organisms were *Staphylococcus epidermidis* (n = 1), *Cutibacterium acnes* (n = 2), *Staphylococcus caprae* (n = 1), and *Staphylococcus capitis* (n = 1). Treatment consisted of intravenous vancomycin (range, 7-14 days) and cefazoline (range, 4-14 days) followed by oral clindamycin for 12 weeks (n = 3), or doxycycline and rifampicin for 11 weeks (n = 1). All patients were free of infection at latest follow-up.

### Subsequent Surgeries and Complications

Four patients required additional surgery to obtain nonunion. All had a history of fracture-related infection. Interestingly, they had negative cultures from the index surgery. Nevertheless, cultures taken during revision surgery were positive for 2 patients (*S epidermidis*, 4 of 5 cultures [case 2], and *S epidermidis*, 1 of 5 cultures [case 4]). Although not proven, the persistence of nonunion may be related to this “surprise” infection.^10^ Both cases healed after revision fixation, antibiotics, and bone grafting. In the other 2 cases, revision fixation with bone grafting led to uneventful healing. Revision surgery included blade plate exchange (cases 2 and 5) and additional 3.5 locking compression plates (cases 3 and 4). At final follow-up, all 4 patients were full weightbearing.

There were 2 additional minor complications. In 1 patient, 3 screws were noted to be broken 1 year after index surgery. Hardware removal was not necessary as union was already achieved. One patient had lower leg hypoesthesia and inability to extend the hallux, most likely because of a nerve stretching due to surgery.

Two patients requested removal of hardware at 3 and 5 years, respectively, because of local irritation.

### Patient-Reported Outcome Measures (n = 13)

Median PCS was 38 (IQR, 34-48, range 17-58, *P* = .009) and MCS was 52 (IQR, 45-60, range 33-62, *P* = .701). Hence, the physical score was significantly worse as compared to the reference population, whereas the mental score did not differ.^1^

Median FAOS was 73 (IQR, 48-83) with median subscores for pain 97 (IQR, 58-100), symptoms and stiffness 61 (IQR, 50-75), daily functioning 84 (IQR, 49-96), sport and recreation 25 (IQR, 15-60), and for quality of life 38 (IQR, 19-50).

## Discussion

Poor bone stock, malalignment, stiffness, scarring, and poor soft tissues from previous surgeries require careful planning when treating a nonunion or failed arthrodesis around the ankle. In this cohort of 15 patients with challenging peri ankle bone healing problems, we were able to eventually achieve union with meticulous debridement and use of a blade plate with autogenous grafting. Additional surgery was required in 4 of 15 patients to obtain union. Our patient-reported outcomes showed fair function at final follow-up.

Alternative fixation methods for a nonunion around the ankle are numerous, including standard or locked plating, external fixation, intramedullary nailing (IMN), bone transport, or arthrodesis.^2,15,25,28^ Reed and Mormino^25^ reviewed treatment options for distal metaphyseal tibial nonunions. The Ilizarov frame allows soft tissue preservation, compression, and correction of malalignment. However, the frame is considered unpleasant and pin tract infections are common.^11,28^ The advantage of IMN is that it disrupts less soft tissue and provides internal bone grafting by reaming. However, in case of malalignment, correction of deformity may require Poller screws. Also, obtaining compression across an oblique nonunion with an IMN is difficult.^29^ The introduction of locking plates has increased options for distal tibial nonunion or a failed arthrodesis.

To circumvent these problems, we often use a blade plate in a periarticular nonunion. The main advantage is that the blade has a small footprint in the often osteopenic and small distal fragment. The inserted material-volume using a blade plate is equal to locked compression plating but in our opinion allows better axial and rotational compression and fixation. In our location, the cost of a blade plate was 77% less than the cost of a locking compression plate.

Several studies evaluated the use of a blade plate in the distal tibia and ankle (Table 2).^3,8,9,12,14,16,23,24,26,32^ Most use the blade plate as a primary arthrodesis method in comminuted pilon fractures, diabetic arthropathy, or severe cases of osteoarthritis.^3,9,12,16,24,31,32^ In a review by Gross et al,^13^ methods of salvage ankle arthrodesis were compared after failed total ankle replacement. The blade plate and bone graft fusion technique resulted in the highest union percentage at first attempt with few complications.

**Table 2.** Angled Blade Plate Fixation for Problems Around the Ankle.

Study	Type of Study	No. of Patients	Indication(s) for Surgery	Surgical Approach	PROMS	Follow-up (mo)	Time to Fusion (mo)	Healing Rate	Complications
Gruen and Mears^14^	Retrospective, single center	5	Nonunion of the distal tibial metaphysis, ankle, or subtalar joint	Posterior approach, TTCA, 95-degree blade plate (50-mm)	Clinical grading score. Pre- (13/44) and postoperative (44/48)	52 (range, 24-64)	4 .0 (range, 3-6)	100%	None
Chin et al^8^	Retrospective, single center	13	Distal tibial metaphyseal nonunion and failed TTA	Anteromedial, lateral, and anterolateral approach. DTF or TTA, 90-degree blade plate	None	34.2 (range, 24-55)	3.6 (range, 2.8-4.6)	100%	Screw breakage (2)
Reed and Mormino^26^	Prospective case series, single center	11	Distal tibial metaphyseal nonunion	Posteromedial approach, DTF, 90-degree blade plate	AOFAS pre- (29/100) and postoperative (89/100)	11, (range, 5-33)	2.8 (range, 2.3-4.6)	100%	Deep infection (1)
Gorman et al^12^	Retrospective, single center	40	Tibiotalar or subtalar osteoarthritis, failed TTCA, nonunion, malunion	Posterior approach, primary/revision TTA or TTCA, 90-degree blade plate (35-50-mm)	None	47 (range, 14-137)	<6.0 (n=29), 6-12 (n=4)	83%	18 major; nonunion (7), delayed union (4), deep infection (4), severe pain (2), DVT (1) 8 minor; superficial infection (1), delayed wound healing (3), peripheral neuritis (1), stress fracture (2), hardware failure (1)
Zelle et al^32^	Retrospective, single center	17	Primary ankle arthrodesis of nonreconstructable pilon fractures	Posterior approach, TTCA, 95-degree blade plate	Postoperative SF-36 (52/100)	>24	4.3	95%	Nonunion (1), DVT (1), cellulitis (1)
Bozic et al^3^	Retrospective, single center	14	Primary ankle arthrodesis in nonreconstructable pilon fractures	Posterior and lateral approach, TTA, 90-degree blade plate (40-50-mm)	None	12.4 (range, 6.4-23.2)	3.4 (range, 2.3-4.8)	100%	Plate breakage (1), deep infection (1)
Cinar et al^9^	Retrospective, single center	4	Diabetic neuroarthropathy	Posterior approach, TTCA, 95-degree blade plate (50-60-mm)	Clinical grading score. Pre- (17/44) and postoperative (43/48)	24 (range, 12-35)	4.5 (range, 3-6)	75%	Infection (1), fibrous union (1)
Myerson et al^24^	Retrospective, multi center	30	Ankle and hindfoot deformity due to diabetic neuroarthropathy, arthritis or avascular necrosis	Lateral approach, TTCA, 95-degree blade plate	None	48 (range, 19-112)	3.7 (range, 2.8-4.1)	93%	Superficial infection (3), stress fracture (2)
Morgan et al^23^	Retrospective, multi center	6	Delayed union or posttraumatic arthritis after tibial plafond fractures	Posterior and lateral approach, TTA, 90-degree blade plate (30-50-mm)	None	8.0 (range, 5.3-11.2)	6.0 (range, 4.6-7.8)	100%	None
Hanson and Cracchiolo^16^	Retrospective, single center	10	Arthritis	Posterior approach, TTCA, 95-degree blade plate (50-60-mm)	Customized AOFAS pre- (21/86) and postoperative (70/86)	37 (range, 12-71)	3.4 (range, 2.1-6.0)	100%	Tibial nerve neuropraxia (1), DVT (1), plate removal (3)
Wera and Sontich^31^	Retrospective, single center	20	Arthritis	Lateral approach, TTA, custom 90-degree 3.5-mm LCDC plate	Postoperative AOFAS (78/100)	37 (range, 2-96.7)	3.9 (range, 1.3-8.8)	100%	None

Abbreviations: AOFAS, American Orthopaedic Foot & Ankle Society ankle-hindfoot score; DTF, distal tibial fixation; DVT, deep vein thrombosis; LCDC, limited contact dynamic compression; PROMS, patient-reported outcome measurements; SF-36, 36-Item Short Form Health Survey; TTA, tibiotalar arthrodesis; TTCA, tibiotalocalcaneal arthrodesis.

Use of a blade plate as salvage in nonunion around the ankle has been previously described.^8,12,14,26^ One of the first were Gruen and Mears,^14^ using a posterior approach in 5 distal metaphyseal ankle nonunions with a union rate of 100% after an average of 4 months. Chin et al^8^ described the use of a blade plate for nonunion in the metaphyseal tibia or tibiotalar joint using an anteromedial, lateral, or anterolateral approach. Union was achieved in all patients at a mean of 3.6 months. We prefer a posterior approach through the Achilles tendon, as this provides better soft tissue coverage, avoiding the often-thin anterior soft tissue.^27^ Gorman et al^12^ performed a posterior blade plate ankle arthrodesis in a heterogeneous group (n = 40) with a variety of indications for primary or revision arthrodesis. Union was achieved in 83%, with 18 major and 8 minor complications. Reed et al^26^ described 11 distal metaphyseal nonunions treated with blade plate fixation. All patients united after a median of 2.8 months. AOFAS scores improved from 29 to 89. Their cohort consisted of distal tibial fixations, whereas 9 of 15 of our patients had an arthrodesis, which obviously leads to decreased function.

There are limitations to this report. First, the retrospective design lacked predefined methodological outcome assessment and follow-up variables. In addition, only 9 of 15 of patients had follow-up using CT scans.^7^ However, despite the retrospective aspect of our study, we were able to collect many detailed outcomes from the medical record. Second, preoperative patient-reported outcome measurements were not available to compare with the postoperative results. Nevertheless, we believe that the improvement of mobility (ie, less use of assistive devices) suggest an important increase in functional outcome. Third, all patients were treated by a fellowship-trained surgeon with expertise in nonunion treatment, which may limit the generalizability of our results. However, we believe that our detailed surgical description enables surgeons to use this technique.

In conclusion, we found the 95-degree condylar blade plate combined with autogenous bone grafting to be a reliable approach for salvaging nonunion around the ankle. With a small footprint, the blade plate allowed excellent fixation. The combination of aggressive debridement, treatment of associated infection, alignment, compression, and fixation with autogenous (ie, biologically active) bone graft, resulted in a high union rate, and improved mobility. Physical function was diminished compared with normative means. We hope that this technique does not become obsolete for future generations.

## Supplemental Material

sj-pdf-1-fai-10.1177_10711007231165303 – Supplemental material for Blade Plate With Autogenous Bone Grafting to Salvage Peri Ankle Nonunions Click here for additional data file. Supplemental material, sj-pdf-1-fai-10.1177_10711007231165303 for Blade Plate With Autogenous Bone Grafting to Salvage Peri Ankle Nonunions by Mees Paulus Emmelot, Robert Kaspar Wagner, Stein Jasper Janssen and Peter Kloen in Foot & Ankle International
